# Sexual variation in India: A view from the west

**DOI:** 10.4103/0019-5545.69244

**Published:** 2010-01

**Authors:** Gurvinder Kalra, Susham Gupta, Dinesh Bhugra

**Affiliations:** Department of Psychiatry, T.N.M.C and B.Y.L.Nair Charitable Hospital, Mumbai Central, Mumbai - 400 008, India, India; 1 East London NHS Foundation Trust, Assertive Outreach Team - City and Hackney, 26 Shore Road, Hackney, London, E9 7TA, UK; 2 Department of Health Service and Population Research, Institute of Psychiatry, King’s College London, De Crespigny Park, London SE5 8AF, UK

**Keywords:** Bisexuality, hijra, homosexuality, men who have sex with men, sexual variation, sexual fluidity

## Abstract

Sexual variation has been reported across cultures for millennia. Sexual variation deals with those facets of sexual behavior which are not necessarily pathological. It is any given culture that defines what is abnormal and what is deviant. In scriptures, literature and poetry in India same sex love has been described and explained in a number of ways. In this paper we highlight homosexual behavior and the role of hijras in the Indian society, among other variations. These are not mental illnesses and these individuals are not mentally ill. Hence the role of psychiatry and psychiatrists has to be re-evaluated. Attitudes of the society and the individual clinicians may stigmatize these individuals and their behavior patterns. Indian psychiatry in recent times has made some progress in this field in challenging attitudes, but more needs to be done in the 21^st^ century. We review the evidence and the existing literature.

## INTRODUCTION

Sexual variation is sexual behavior which varies from the usual heterosexual intercourse; the behavior includes alternative sexual orientation such as homosexuality and bisexuality. Its description in various forums has been well known across the globe for millennia. A recent term, which has been used extensively, is ‘Men who have Sex with Men’ (MSM). Gender roles and gender role identities may fluctuate, as does the sexual behavior, depending upon the availability of sexual partners and opportunities.

Although the data on sexual variation are extremely limited, especially from India, in this paper we propose to highlight some of the conceptual issues. We aim to describe some of these behavior patterns in historical accounts and raise issues related to managing these in clinical settings.

### Variation

In this paper, we focus on bisexuality and homosexuality. Sexual orientation refers to a person’s preference for sexual and emotional relationships with a particular sex.[1] Sexuality is not just sexual, but also has an emotional component and impact. Sexual behavior should not be equated with sexuality. Societal attitudes influence this behavior and whether individuals carry these out openly or in secret. For bisexuality, the male role takes on a different dimension. In a patriarchal society such as India the roles of fathers, brothers and husbands may be threatened by variations in sexual behavior.

However, sex and gender are often confused. The sex of an individual is determined biologically, whereas gender may be influenced by social factors. Bullough[2] described societies as ‘sex positive’ or ‘sex negative’. Sex positive societies celebrate sexual activity and the sexual act is seen as meant for pleasure, whereas the main function of sex in sex negative societies is seen as for procreation. In addition, a further complicating factor in individual sexual behavior is not only personality and orientation, but also whether they meet social expectations, which are likely to be affected by kinship and socio-centric traditions of the society they come from. India, by and large, remains a traditional socio-centric society rapidly moving to an ego-centric society as a result of urbanization, industrialization under the overall impact of globalization. In this paper we do not propose to cover other variations, such as pedophilia, but the aim is to focus on homosexuality and bisexuality. We will discuss the notion of *hijras* in India, their role in society and associated attitudes. Historically, psychiatry as a profession has placed itself in treating these conditions sometimes primitively and sometimes without adequate safeguards as agents of social control. However, in the West this approach has been discredited, though it continues in parts of the world, including India.

## HISTORY

The attitudes to bisexuality and homosexuality in India have been ambivalent to say the least. From historical accounts, it appears that these types of behavior patterns were tolerated and celebrated. The dilemma for the modern day clinical practitioner in India is whether homosexuality (and which is often equated with homosexual behavior) is a Western concept and was imported into the country with the British *Raj*. Often this view is expressed by politicians on both ends of the political spectrum. Interestingly, it would appear that a large proportion of MSM in India are married, unlike in the West. Whether this reflects true bisexuality or simply homosexual desire subservient to socio-centric society needs to be explored further. These individuals are less likely to see themselves as homosexuals, even in large metropolises. Further, the advent of the internet and access to sexual partners through the web has meant that the attitude and behavior may be becoming more secretive, even in the younger generation.

Early Buddhist and Hindu periods covered in ancient texts such as *Manusmriti, Arthashastra, and Kamasutra* refer to same sex attraction and behavior. The Buddhist tradition, as indicated in the pillar caves of Karle (50-75 CE), shows two bare-breasted women embracing each other. In Hindu scriptures, for example, *Bhagiratha* is born from the union of two women. *Shikhandi* changes gender and *Ardhnarishwar* (half-man, half-woman) are described. *Ayyappa* (dual gendered god) is worshipped and honoured by hijras. Several sculptures and carvings in *Khajuraho* depict same sex behavior, including mutual fellatio and orgiastic scenes. The God *Ayyappa* was born of intercourse between *Shiva* and *Vishnu* when the latter temporarily assumed the form of a beautiful seductive woman-*Mohini*.[3] A number of 14^th^ century texts in Sanskrit and Bengali (including *Krittivasa Ramayana*) narrate how King *Bhagiratha* was born of the union between two women blessed by Lord *Shiva*.[4]

Hijras in India have a degree of importance. They would come and dance in Hindu households at the times of marriage and when a male child is born. They often refer to themselves as those having no (sexual) desire for women.[5] They are differentiated clearly from effeminate or gay men in India.[6] Reddy (2005)[7] notes that, by virtue of their importance, the hijras are man-minus-man, but also man-plus-woman. They wear female attire, use make up, have feminine names and behave in a feminine manner-some more than the others. They may be born as hermaphrodites or castrated.[5] Various sociological studies[8–10] reveal that many people living in these organized hijra communities practice male homosexual prostitution. Some members of this community were found to be permanently attached to prosperous male homosexuals who took care of their financial needs.

In Persian and Sufi traditions, love of a man for another man is described, although it can be argued that it is mortal man loving god. Muslim presence in India raised the notion of poetry in the form of love songs-*ghazals* and the concept of one male’s love for another. Gupta (2008)[11] argues that in *Baburnama*, the Mughal Emperor Babur is quite clear about his indifferent love for his wife and his preference for a young man. It is quite possible that such attachments were those of a mentor and guide in an emotional sense rather than a sexual one. Many paintings and works of fiction dating from several centuries depict same sex love and loss. For example, in Siraj Aurangabadi’s poem *Bustani-i-Khayal* the narrator, heart broken over the loss of his (male) beloved, seeks solace in the company of courtesans who cheer him up.[12] Many poets used feminine pen names to hide their true desire in response to British Victorian attitudes to homosexuality. Although selective, the brief review indicates that same sex relationships, whether emotional attachments or sexual, have been around in India for a considerable period of time and are not an import from the West. Recent studies by several authors such as Vanita and Kidwai[4] confirm these.

## ATTITUDES

There is little doubt that negative societal attitude to homosexuality has contributed to medicalization of the variation leading to often inappropriate interventions. Foucault (1988, 1990)[13] saw this control as another form of social control over an individual’s sexual expression. He notes that this is not dissimilar to canonical law and points out that any treatment should be looked through the lens of human rights.

Homosexuality was removed from the Diagnostic and Statistical Manual in the 1970s and in the other diagnostic system ICD-10 only ego dystonic homosexuality is included. The British made male homosexual behavior illegal and it is rather ironic that 62 years after independence archaic colonial laws such as Section 377 of the Indian penal code still exist. Although the Delhi High Court has termed the Act illegal, pending appeals to the Supreme Court make its total repeal and amendment to the law delayed. It is also interesting that male homosexual behavior is illegal, but not the female one. In spite of the evidence to the contrary and appeals by various eminent Indians, including the Nobel prize winner Professor Amartya Sen, and the fact that the same law was repealed in Britain in 1967, India remains at par with countries such as Zimbabwe! Attitudes to male homosexuality are often negative, especially because in patriarchal societies heterosexual males may feel threatened. These are negative because of extra-marital affairs being seen as sinful and dirty. Furthermore, quite wrongly, homosexual behavior is equated with pedophilia, which is seen as a form of recruitment into homosexuality.

### Implications of attitudes

Thus these negative attitudes are influenced by legal factors and stigma related to ‘non-standard’ sexual behavior. As is evidenced from several countries in Africa, some politicians believe that homosexual behavior is single handedly responsible for the spread of HIV infections and AIDS, thereby denying potentially life-saving medications and preventive education. In addition, religious dogma is often brought in to emphasize the notion of ‘the other’, thus creating a further sense of alienation. What is surprising is how attitudes to female homosexual behavior vary from those towards males. Female same-sex activity is seen as arousing by the males. Bisexual individuals are often accused of ‘sitting on the fence’ or ‘being unable to make their mind up’ or ‘having the best of both worlds’. It is also likely that measurement of sexual orientation is often rather vague and uncertain. Kinsey (1948)[14] developed his scale and used it on a large number of people; the questions focused on whether individuals saw themselves as predominantly homosexual or heterosexual. However, no distinction is made between same sex or opposite sex fantasy, arousal or behavior. Klein’s grid test (1993)[15] measures a person’s sexual orientation on a continuum between straight and gay giving a more subtle and nuanced measurement of sexual identity. However, the use of terms such as straight or gay is also problematic. ‘Gay’ denotes a political position, while ‘homosexual’ identifies with being content with who they are and taking this position publicly. ‘Straight’ is used in contrast to ‘bent’, which is a pejorative term.

Kinsey (1948)[14] in his study reported that 4% were exclusively homosexual and 46% were somewhere between being exclusively homosexual or heterosexual. These findings have been taken for granted and are often used both by anti-gay and pro-gay lobbyists.

LeVay and Baldwin (2009)[16] note that sex is about identity as well as relationships. They also argue that the prevalence of bisexuality depends upon definitions being used (p 484). Sometimes questions are raised about the validity of this behavior. Malteson (1991)[17] points out that a bisexual male may have different emotional or physical attachments to males or females.

Bisexuality is an under-researched area in human sexuality. It is also seen as a somewhat grey area in our understanding of sexuality and encompasses sexual orientation, identity and behavior. Even though some studies have been conducted over the years following Kinsey’s influential era, they have been unable to clarify many of the aspects of bisexuality and bisexual behavior. Research in bisexual sexual behavior received attention after the HIV epidemic became evident and role of bisexual men as a potential ‘bridging group’ between the genders was considered for possible interventions to reduce HIV transmission.

Defining and conceptualizing bisexuality is not straight-forward. There is a distinction between bisexual identity and behavior. Bisexual behavior may be more common than people identifying themselves as ‘bisexual’. A wide range of sexual identities may accompany bisexual behavior. Sexual identities are also linked to gender identities, situational, cultural and environmental factors (men in prison etc). There is usually an asymmetry of practice, with sexual activities with one gender predominating with possible temporal variations. The variations in level of erotic desire and identity are less well-known and certain ‘fluidity’ is likely. An individual may see himself as heterosexual but their fantasy or behavior may be homosexual depending upon circumstances.

Development of sexuality is both multi-factorial and multi-faceted, with the interplay of various biological (e.g., genetic, hormonal, physical), physiological (e.g., genital arousal), psychological (e.g., psychodynamic, behavioral, cognitive-behavioral) developmental processes over the years. Sexual behavior is also dependent on psychological (e.g., attraction, desire, fantasy, eroticism, romantic), behavioral, physiological (arousal), emotional and social (e.g., social acceptance, self-identity, sexual politics) aspects. As our understanding of human behavior, psycho-social and biological development and brain functioning has improved, there is greater effort to integrate this knowledge to find an explanation of human sexual behavior. The sexual arousal pattern in bisexuals is not well-known. Understanding sexual arousal pattern in bisexuals is likely to provide better insight into intra-group range and diversity (if any) and how it differs from homosexual and heterosexual arousal patterns. This in turn could shed further light into the relationship between physiological functioning (e.g., arousal) and bisexual behavior and identity.

There has been a great deal of argument over whether sexuality is about sexual *preference* or sexual *orientation*. In the general dearth of studies in bisexuality, the studies of the brain structures, brain functioning, developmental aspects, behavioral, arousal pattern and mental health of homosexuals (mostly compared to heterosexuals) can provide valuable information in these areas as well as methodological designs.

### Identity

The process of development and sexual identity formation may not follow a single pattern; sexual identity can be fluid and will depend upon what the external reference points are. A recent US study published in the Psychological Science on the debate of male bisexuality by Gerulf Rieger *et al*.[18] found that self-reported sexual arousal (to both male and female sexual stimuli) differed from genital arousal patterns, which were more strongly associated with one sex or the other (most of the time the pattern being similar to gay men). They concluded that ‘male bisexuality is not simply the sum of, or the intermediate between, heterosexual and homosexual orientation. Indeed, with respect to sexual arousal and attraction, it remains to be shown that male bisexuality exists’. These findings were deemed controversial and drew mixed reactions from various groups. The study was criticized as being too simplistic/reductionist and not taking into account emotional and sensual aspects of sexuality.

There are no comparative data available from India, although there have been very few case studies done prior to the de-medicalization of homosexuality by the APA.[19] From a sex therapy clinic in north India, Verma *et al*.[20] reported less than 5% of attendees having had homosexual contact. A slightly lesser figure of 3% homosexual contact and 5% bisexual contact has been reported by Kalra and Kamath (2009)[21] in an unpublished study. Bhugra *et al*.(1997a)[22] and Bhugra (1997b)[23] studied coming out in South Asian gay men in the UK and western India, and found that persons most likely to come out were generally their friends. Family played a significant role in the lives of gay men, who found it very difficult to come out to their families. Interestingly, in another study Bhugra *et al*[24] found that sexual fantasy in gay men in Mumbai was largely same sex, compared with heterosexual men.

## ISSUES IN MANAGEMENT

As noted above, the epidemiological data on prevalence of homosexuality and bisexuality in India are lacking, although a recent survey in *India Today*[25] revealed that in the northwestern Indian city of Jaipur, 15% of men had sex with men. In Hyderabad, 61% approved of homosexuality. In Ahmedabad, in 2006, 56% reported having had a homosexual experience.

However, there is plenty of anecdotal evidence; even in metropolitan cities in India, psychiatrists use aversion therapies to change sexual orientation. It could be argued that this is simply a reflection of what Weeks (1981)[26] has already pointed out ‘…individuals are very much alike sexually and that it is an equally simple matter for all of them to confine their behavior to the single patterns which the (social) mores dictate’. Therapies used to change the orientation (or is it simply the behavior that is being changed?) arguably are ethically questionable.[27] The challenge for the therapists also is - where does bisexuality and bisexual behavior fit in all of this? Individuals may be pushed to seek treatment for their alternative sexualities because of family pressures to get married and produce heirs. Early studies reported treatment patterns in India mostly using behavioral approaches.[28,29] The distinction between ego dystonic alternative sexualities and ego syntonic ones need to be clarified and addressed.

The challenge for Indian psychiatry is not to isolate itself from global psychiatry but within the specific cultural context develop treatment guidelines which are clear and helpful. High levels of homosexual experiences do not translate necessarily into homosexuality or help-seeking. In the survey noted above, of those who admitted to having participated in an orgy, 52% of males had a male partner with them. This again confirms that there is a massive degree of variation in the community and the psychiatric profession needs to acknowledge this and take this into account. Overall, 12% of men and 6% of women had participated in orgies. Although the main emphasis was on understanding sexual desire and fantasies, it was largely based in cities. The important factor that all psychiatrists have to bear in mind is that their task is not only to be guided by the society, taking into account the cultural context, but it is essential that as opinion formers and leaders they educate the society and dispel myths. The fact that homosexual behavior has existed for centuries indicates that the ‘normal variation’ is part of human nature. A lack of space does not allow us to develop themes related to evolutionary psychiatry. It is sufficient to say that alternative sexualities exist and do influence social mores. It is important that young adults who may be struggling to deal with their sexual orientation and behavior are not branded psychiatrically ill and treated for it.

## CONCLUSIONS

In recent times in the West, there has been a shift from seeing alternative sexualities as sinful or criminal activity. In some countries, criminal activity has given way to a grudging social acceptance. For example, in the UK, homosexual behavior was a crime till 1967 and then came a change in the age of consent and civil partnerships, which have produced a grudging acceptance. Various universities and academic institutions run courses on queer theory. The word of abuse ‘queer’ has been re-appropriated as a symbol of pride and points towards a sexuality which is more fluid. Sexual fluidity rejects the attempts of society to force everybody into pigeonholes and standardize sexual behavior. Similarly, in recent times various Presidents of the Indian Psychiatric Society have challenged the age old assumptions.[30,31]

Sathyanarayana Rao and Avasthi (2008)[32] have outlined a road map for sexual medicine from a psychiatric perspective; all these authors indicate a change in the views of psychiatrists to treatment of homosexuality. As agents of social change, Indian psychiatrists need to lead the charge in persuading society to accept sexual diversity and alternative patterns of sexual desire.

## References

[1] Weiten W, Weiten W (1995). Motivation and Emotion. In: Weiten W Psychology: Themes and Variations.

[2] Bullough V (1976). Sexual Variation in Society and History.

[3] Conner RP, Sparks DH, Sparks M (1997). Cassell Encyclopaedia of Queer Myth, Symbol and Spirit.

[4] Vanita R, Kidwai S (2000). Same Sex Love in India.

[5] Rowland DL, Inrcrocci L (2008). Handbook of Sexual and Gender Identity Disorders. NJ, John Wiley and Sons.

[6] Cohen L, P Abramson, S Pinkerton (1995). The pleasures of castration. Sexual Nature, Sexual Culture.

[7] Reddy G (2005). With Respect to Sex: Negotiating Hijra Identity in South India.

[8] Idnani A (1970). A study of Hijras- Delhi. Dissertation submitted for M.A. Degree in Social Work.

[9] Nerurkar N (1971). A Study of Hijras- Hyderabad. Dissertation for M.A. in Social Work.

[10] Goswami V (1973). A study of Hijras- Agra. Dissertation submitted for M.A. in Social Work.

[11] Gupta S (2008). Portrayal of homosexuality in India arts. Presented at ANCIPS, Kolkata.

[12] Sarvari AQ (1998). Kulliyat-i-Siraj.

[13] Foucault M (1988, 1990): History of Sexuality (vols 1, 2 and 3).

[14] Kinsey (1948). Sexual Behaviour in the Human Male.

[15] Klein F, F Klein (1993). Sexual Orientation Grid Test. The Bisexual Option.

[16] LeVay S, Baldwin J (2009). Human Sexuality. Sunderland MA, Sinauer Associates.

[17] Malteson D, L Hutchins, L Kashmanu (1991). Bisexual feminist men. Bi Another Name: Bisexual People Speak Out.

[18] Rieger G, Chivers ML, Bailey JM (2005). Sexual arousal patterns of bisexual men. Psychol Sci.

[19] Pradhan PV, Ayyar KS, Bagadia VN (1982). Male homosexuality: A Psychiatric Study of Thirteen Cases. Indian J Psychiatry.

[20] Verma KK, Khaitan BK, Singh OP (1998). The frequency of sexual dysfunctions in patients attending a sex therapy clinic in north India. Arch Sex Behav.

[21] Kalra GS, Kamath R Psychosocial profile of male patients presenting with sexual dysfunction. Unpublished MD dissertation 2009, Maharashtra University of Health Sciences, Nashik.

[22] Bhugra D (1997). Coming out by Asian gay men in the United Kingdom. Arch Sex Behav.

[23] Bhugra D (1997). Experiences of being a gay man in urban India: A descriptive study. Sexual and Marital Therapy.

[24] Bhugra D, Rahman Q, Bhintade R (2006). Sexual fantasy in gay men in India: A comparison with heterosexual men. Sexual and Relationship Therapy.

[25] Bamzai K (2009). Mind Games. In: India Today.

[26] Weeks J (1981). Sexuality.

[27] Narrain A (2004). Queer: Despised sexuality, law and social change.

[28] Sakthivel LM, Rangaswami K, Jayaraman TN (1979). Treatment of Homosexuality by Anticipatory Avoidance Conditioning Technique. Indian J Psychaitry.

[29] Pradhan PV, Ayyar KS, Bagadia VN (1982). Homosexuality: Treatment by Behaviour Modification. Indian J Psychiatry.

[30] Reddy IR (2007). Making Psychiatry a household word. Indian J Psychiatry.

[31] Mohandas E (2009). Roadmap to Indian Psychaitry. Indian J Psychiatry.

[32] Sathyanarayana Rao TS, Avasthi A (2008). Roadmap for sexual medicine: Agenda for Indian Psychiatric Society. Indian J Psychiatry.

